# Opportunistic osteoporosis assessment from routine CT—effect of intravenous contrast agents on absolute values, *T*-scores, and derived classifications in single- and dual-energy CT

**DOI:** 10.1007/s00330-025-11988-1

**Published:** 2025-09-11

**Authors:** Jennifer Gotta, Vitali Koch, Scherwin Mahmoudi, Simon S. Martin, Jan Erik Scholtz, Christian Booz, Katrin Eichler, Simon Bernatz, Philipp Reschke, Tatjana Gruber-Rouh, Tommaso D’Angelo, Thomas J. Vogl, Leon D. Gruenewald

**Affiliations:** 1 Department of Radiology, Goethe University Hospital Frankfurt, Frankfurt am Main, Germany; 2 Department of Biomedical Sciences and Morphological and Functional Imaging, University Hospital Messina, Messina, Italy

**Keywords:** Body composition, Computed tomography, Osteoporosis, Trabecular bone, Contrast media

## Abstract

**Objectives:**

Computed tomography (CT) is widely used for bone health assessment, impacting osteoporosis diagnosis and treatment. However, the influence of intravenous contrast agents on CT-based bone mineral density (BMD) measurements remains debated. This study evaluates the effect of contrast agents on Hounsfield measurements, *T*-scores, and *Z*-scores, assessing their impact on diagnostic accuracy to reduce misclassification and optimize CT-based BMD assessment.

**Materials and methods:**

A retrospective analysis of 597 patients (median age: 66 years, 157 females, 440 males) was performed using dual-energy CT (DECT) scans of the abdomen and chest. All patients underwent non-contrast, arterial, and venous phase CT. Automated segmentation (nnU-Net) delineated L1 and L1–L4 trabecular bone, validated by two radiologists. *T*-scores were calculated according to DEXA-equivalent guidelines.

**Results:**

Based on non-contrast CT, 35% were diagnosed with osteoporosis, 46% with osteopenia, and 18% had normal bone status. Median *T*-score was −2.0 (L1) and −2.1 (L1–L4) (*p* < 0.001). Contrast agents significantly altered BMD values, with median changes of 22.9% (arterial) and 20.1% (venous). The most pronounced changes occurred in patients under 50 years (+99% at L1, *p* < 0.001). In older females, 21% were misclassified as osteopenic instead of osteoporotic (*p* < 0.001).

**Conclusions:**

Contrast agents significantly affect BMD measurements, leading to diagnostic misclassification. This effect should be considered when using CT for osteoporosis diagnosis and treatment planning.

**Key Points:**

***Question*** *Standard CT scans with contrast media may distort bone density measurements, potentially leading to misdiagnosis of osteoporosis and inappropriate clinical decisions*.

***Findings*** *Contrast-enhanced CT scans significantly alter T- and Z-scores, leading to diagnostic shifts in over 50% of patients, especially women over 50*.

***Clinical relevance*** *Our findings highlight the risk of osteoporosis misclassification due to contrast agents in CT imaging, underscoring the need for adjusted interpretation protocols to ensure accurate diagnosis and appropriate treatment, particularly in older adults and female patients*.

**Graphical Abstract:**

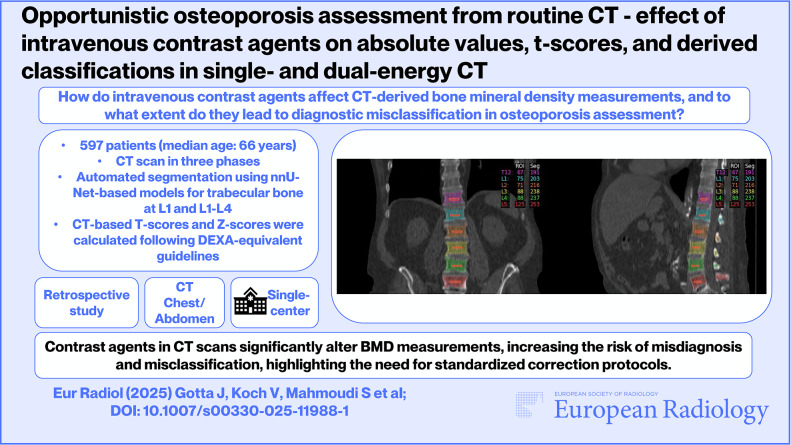

## Introduction

Osteoporosis is a major global health issue, affecting millions of people worldwide and leading to a significant burden on both personal and economic health systems. The incidence of osteoporosis has steadily increased over the past few decades, and this trend is expected to continue as the global population ages [1]. Osteoporosis not only contributes to a decline in physical function but also increases the risk of fractures, with considerable implications for patients’ quality of life and healthcare costs [2].

Accurate evaluation of bone health is essential for diagnosing conditions such as osteoporosis and osteopenia, assessing fracture risk, and monitoring the effectiveness of treatment interventions [3–5]. The most commonly used diagnostic method is dual-energy X-ray absorptiometry (DEXA), which provides areal bone mineral density (BMD) measurements and is widely accepted in clinical practice. However, DEXA has limitations, particularly regarding its precision in distinguishing trabecular from cortical bone and its reduced applicability across diverse patient populations due to factors such as obesity [6]. An alternative approach is quantitative CT (QCT), which offers a more accurate volumetric assessment of trabecular BMD, but it generally cannot be applied retrospectively due to the need for proper positioning and calibration phantoms during the examination [7].

In contrast, advanced imaging techniques such as CT and magnetic resonance imaging (MRI) have shown significant potential for analyzing body composition, including bone composition and density [8–10]. MRI is particularly valuable for evaluating bone marrow composition, microarchitecture, and soft tissue differentiation without exposure to ionizing radiation. However, its application in routine BMD assessment remains limited due to higher cost, longer scan times, and lower accessibility compared to CT [11, 12]. CT scans are increasingly utilized in clinical practice for various purposes, including the evaluation of cancer treatment and the investigation of suspected complications in critically ill patients. These scans often include the spine and provide an opportunity for retrospective analysis of bone density and composition, so non-quantitative methods to estimate BMD, such as simple Hounsfield Units (HU) measurements, are gaining increased attention. Additionally, other studies have shown that automated quantitative assessments of bone, muscle, and fat in contrast-enhanced abdominal CT scans can be reliably correlated with measurements obtained from non-contrast scans [13].

Therefore, this study delves into the significance of contrast enhancement in CT bone density analysis. It examines how varying contrast phases influence the precision of bone density measurements, aiming to optimize imaging protocols for enhanced clinical diagnostics.

## Materials and methods

### Study population

This study retrospectively analyzed dual-energy CT (DECT) and single-energy CT scans of the abdomen and chest, performed for suspected vascular pathologies like aneurysm or aortic dissection, in patients treated at the University Hospital Frankfurt between 17th December 2006 and 27th June 2024. The scans were identified using the internal PACS system.

The inclusion criteria were as follows: patients older than 18 years, with CT scans available across multiple contrast phases, including an unenhanced phase, an arterial phase, and a venous phase. Each scan had to cover at least the abdomen and thorax. The CT scans were analyzed by comparing images obtained from the same patient during the same examination, using identical time points and scanner settings.

Exclusion criteria included incomplete scanner protocols, patients under 18 years of age, or the absence of a CT scan that covered at least the abdomen and thorax.

Approval for this study was obtained from the local ethical committee (ethical approval number: 2024-1854), and the research was conducted in adherence to these guidelines. All analyses were performed following local data protection regulations.

### CT scan protocol

CT examinations were performed on a third-generation dual-source CT device in dual-energy mode (SOMATOM Force, Siemens Healthineers) and a single-energy CT with a sliding gantry (Siemens Definition with sliding gantry). Image series were collected in a craniocaudal direction with a slice thickness of 3 mm. Noncontrast images, bolus-triggered arterial phase images (threshold: 150 HU, scan-delay: 7 s post-trigger and delayed phase images (scan-delay: 45 s post-trigger) were obtained. The volume of iodine-based contrast agent (Imeron 400 with a concentration of 400 mg iodine/mL, Bracco S.p.A.) was adapted to the patient’s body weight to achieve a final concentration of 1.5 mL/kg body weight, resulting in a volume of 90 mL in most cases. The injection was performed using a power injector (Bayer MEDRAD Centargo Bayer AG) at a rate of 4 mL/s. The scans were performed either ECG-triggered or in high-pitch mode. Automatic exposure control systems were activated in all cases. The reference tube current–time product was set to 100 mAs, while the tube voltage was dynamically adjusted by the system, typically ranging between 80 kV and 120 kV depending on patient size and tissue attenuation.

### Automated segmentation and BMD estimation

**Fig. 1:**
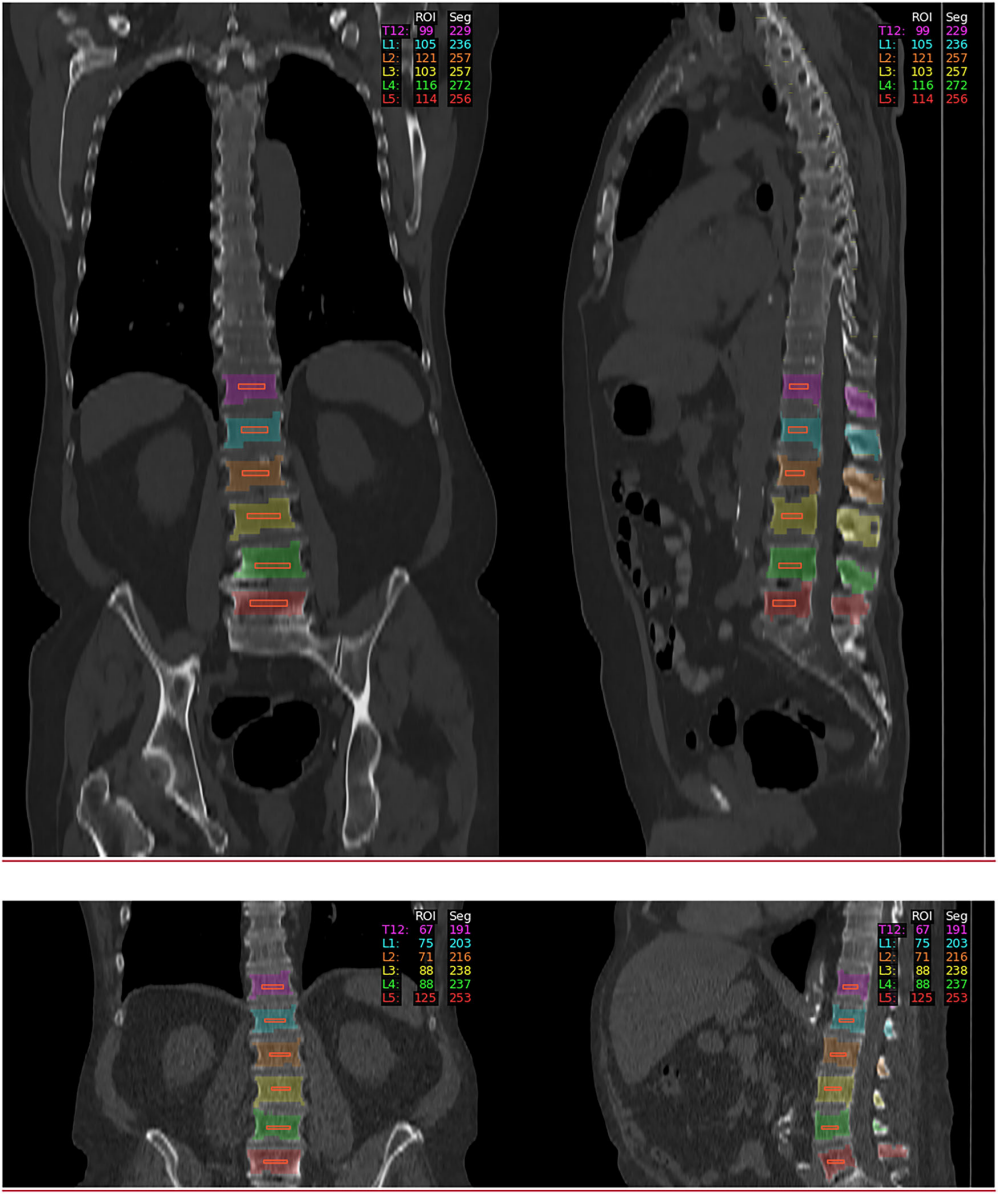
Exemplary automatic segmentation of the spine using publicly available nnU-Net-based models

Image segmentation was performed using nnU-Net-based models [14–17]. Briefly, a region of interest containing the trabecular bone of the vertebral bodies of L1, as well as the trabecular bone of the L1–L4 vertebral bodies, was created by computing the left-right and superior-inferior center of the vertebral body. For each vertebral body, the mean and median trabecular HU were extracted from the created region of interest together with the segmentation volume. This process was performed for all phases in each patient. Images of the created segmentation maps and regions of interest were exported and manually inspected by two experienced radiologists to confirm correct anatomical identification and alignment between the image series. Only datasets in which both radiologists independently confirmed the anatomical correctness of the segmentation were included in the study. In cases of disagreement, the dataset was excluded from further analysis. Figure 1 illustrates an exemplary segmentation.

### *T*-score calculation

CT-based *T*-scores were calculated analogously to DXA-based *T*-scores, following established guidelines [14, 18, 19], but without employing DXA as a direct reference standard. Briefly, the *T*-score is calculated by subtracting the young normal mean trabecular HU, which refers to the average trabecular HU of a 30-year-old person of the same gender, from the patient’s trabecular HU, and then dividing by the standard deviation (SD) of the young normal group’s trabecular HU. Reference values were obtained from our own in-house data, based on 25,000 scans from an ethnically homogeneous cohort. A *T*-score greater than −1.0 indicates normal bone mass. A *T*-score between −1.0 and −2.5 indicates osteopenia. A *T*-score less than −2.5 indicates osteoporosis.

### Statistical analysis

Statistical analysis was performed using R statistical software (R Foundation for Statistical Computing, Vienna, Austria; Version 2023.06.0 + 421) and Python (Version 3.12.6). Normally distributed data were presented as mean ± SD, while non-normally distributed data were reported as median and interquartile range (IQR). Differences in baseline characteristics and changes in *T*-scores and *Z*-scores at the L1 vertebral level, as well as the trabecular bone of the L1–L4 vertebral bodies, were assessed using t-tests, if applicable, or Mann–Whitney tests, Chi-Squared tests, and Kruskal–Wallis tests. A *p*-value < 0.05 was regarded as statistically significant. For non-normally distributed data, the Wilcoxon signed-rank test was applied to assess changes in diagnostic classifications. Additionally, chi-squared tests were used to assess differences between gender groups. A *p*-value < 0.05 was regarded as statistically significant.

## Results

### Patient characteristics

Of the 1021 patients considered for inclusion in this study, 424 were excluded due to incomplete examination protocols.

The final study population comprised 597 patients (median age, 66 years [IQR, 56–76 years]; 157 females, 440 males). Among individuals younger than 50 years (*n* = 78), the median age was 41 years (IQR 35–47 years), while those 50 years and older (*n* = 519) had a median age of 69 years (IQR 60–78 years). All individuals below 50 years were predominantly male (61 males, 17 females). Among participants aged 50 years and above, 140 were females with a median age of 72 years (IQR 63–80 years), whereas 379 were males with a median age of 68 years (IQR 60–76 years). These data highlight a predominantly male cohort with an older median age in the female subgroup aged 50 years and above. Based on non-contrast CT scans, 210 patients (35%) were diagnosed with osteoporosis, 27 (47%) patients with osteopenia, and 108 patients (18%) had normal bone status. In three cases, the *T*-score could not be calculated. Detailed information is given in Table 1.

**Table 1:** Baseline characteristics of the study cohort

Variables	Total (*n* = 597)	Females (*n* = 157)	Males (*n* = 440)	Total < 50 years (*n* = 78)	Total > 50 years (*n* = 519)	Females > 50 years (*n* = 140)	Males > 50 years (*n* = 379)	*p*-value
Age (years)	66 (56–76)	70 (59–79)	65 (56–75)	41 (35–47)	69 (60–78)	72 (63–80)	68 (60–76)	0.406
Sex								
Male	440	0	440	61	379	0	379	0.001
Female	157	157	0	17	140	140	00	0.001
Diagnosis								< 0.001
Healthy	108	16	92	41	67	8	59	
Osteopenia	276	37	239	34	242	28	214	< 0.001
Osteoporosis	210	104	106	0	210	104	106	< 0.001
Unknown	3	1	2	0	3	1	2	–

### Variability in trabecular HU measurements across different CT phases

Based on non-contrast CT scans, the median HU of the trabecular bone of L1 was 127.7 (IQR 5.7–162.6) and the median HU of the trabecular bone of L1–L4 was 121.8 (IQR 89.99–156.93) (Supplementary Tables 1, 2, 7, and 8).

**Fig. 2:**
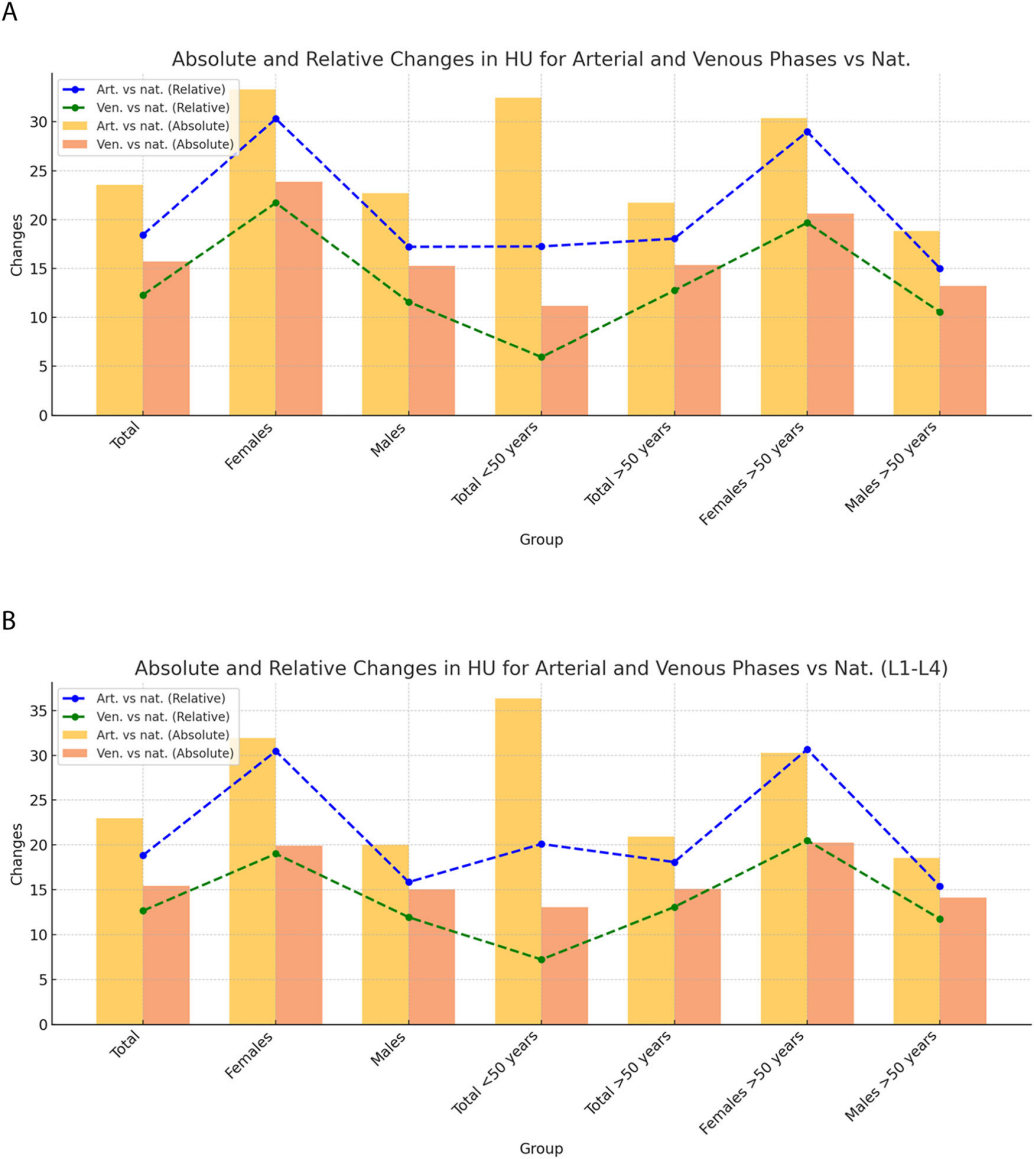
Absolute and relative changes of HU of the arterial and venous phase compared to non-contrast

The median increase in HU of the trabecular bone of L1 between unenhanced examinations and examinations performed in the early arterial phase was +23.6 HU (+18.5%) (*p* < 0.01) and +15.7 HU (12.3%) in the delayed phase (*p* < 0.001) with higher changes observed in female patients (+33.3 HU, 30.3%) (*p* < 0.001) (Fig. 2A).

A similar pattern was observed in the HU of the trabecular bone of L1–L4 with a median increase of +23.0 HU (18.9%) in the arterial phase (*p* < 0.001) and +15.4 HU (12.7%) in the delayed phase (*p* < 0.001) (Fig. 2B).

### Comparative analysis of *T*-score changes across different CT series

**Fig. 3:**
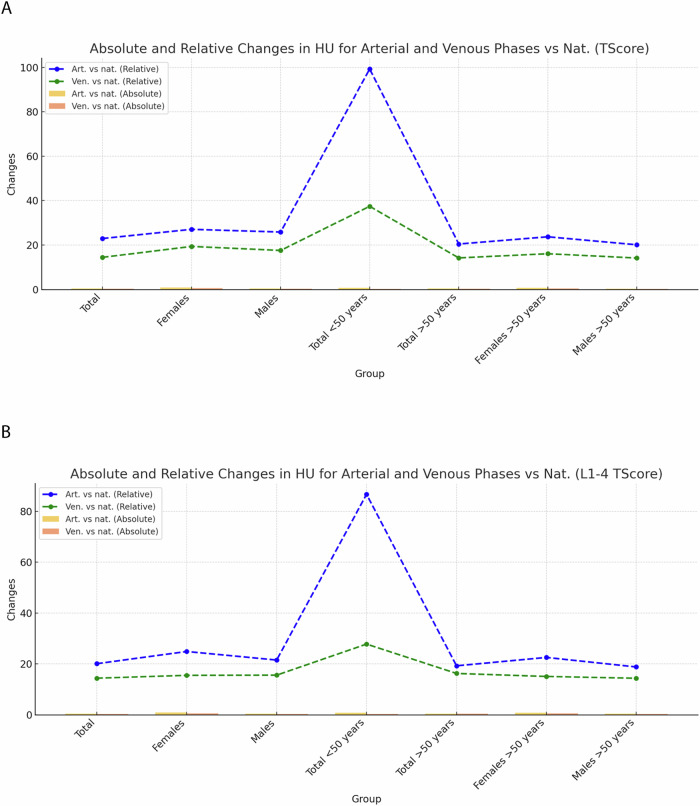
Absolute and relative changes of the *T*-score of the arterial and venous phase compared to the non-contrast

Analysis of trabecular *T*-scores at the L1 vertebral level and at the level L1–L4 in the non-contrast-phase revealed a median *T*-score of −2.0 (IQR: −2.82 to −1.29) (*p* < 0.001) for the whole patient cohort at the L1 vertebral level and a median *T*-score of −2.1 (IQR: −2.87 to −1.48) at the level L1–4 (*p* < 0.001) with a median change of 0.47 (22.9%) between unenhanced examinations and examinations performed in the early arterial phase (*p* < 0.01) and 0.43 (20.1%) in the delayed phase (*p* < 0.001) with significant higher changes observed in patients under 50 years between unenhanced examinations and examinations performed in the early arterial phase with +0.71 (99%) for L1 and +0.78, (86.7%) for L1–4, respectively (*p* < 0.001) (Fig. 3A, B and Supplementary Tables 3, 4, 9, and 10).

### Comparative analysis of *Z*-score changes across different CT series

Analysis of trabecular *Z*-scores at the L1 vertebral level and at the level L1–4 in the non-contrast-phase revealed a median *Z*-score of −0.54 (IQR: −1.13 to 0.17) (*p* = 0.01) for the whole patient cohort at the L1 vertebral level and a median *Z*-score of −0.63 (IQR: −1.21 to 0.01) at the level L1–4) *p* = 0.01) with a median change of 0.62 (115.9%) between unenhanced examinations and examinations performed in the early arterial phase (*p* < 0.01) and 0.39 (71.7%) in the delayed phase (*p* < 0.001) at the L1 level and 0.55 (88.2%) in the arterial phase and 0.37 (58.8%) at the level L1–4.

**Fig. 4:**
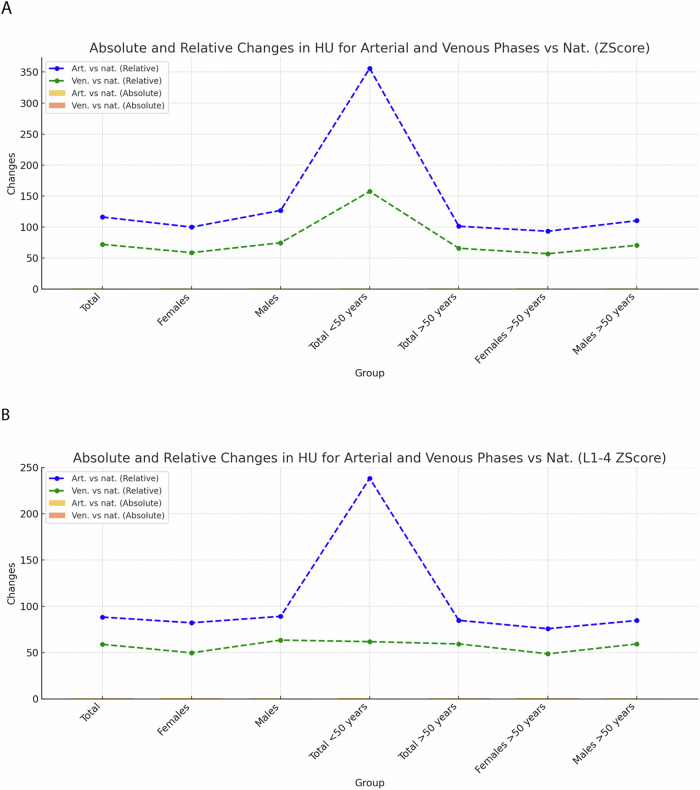
Absolute and relative changes of the *Z*-score of the arterial and venous phase compared to the non-contrast

Significantly higher changes were observed in patients under 50 years between unenhanced examinations and examinations performed in the early arterial phase with +0.77 (355.9%) for L1 and +0.94, (238.2%) for L1–4, respectively (*p* < 0.001) (Fig. 4A, B and Supplementary Tables 5, 6, 11, and 12).

### Diagnostic impact of different CT series

Of the 597 patients in total, 309 experienced a diagnosis change, representing 51.76% of the overall study population. Specifically, 29.31% of patients showed a diagnosis change from the non-contrast phase at the L1-level to the arterial phase, while 22.45% experienced a change to the venous phase.

The most common misclassifications when comparing non-contrast to arterial phases were from Osteopenia to a normal bone status with 97 patients (16%) (*p* < 0.001) and from Osteoporosis to Osteopenia with 72 patients (12%) (*p* < 0.001).

In the comparison between non-contrast and venous phases, the most common misclassifications were also from Osteopenia to a normal bone status with 48 patients (8%) (*p* < 0.001) and from Osteoporosis to Osteopenia with 80 patients (13%) (*p* < 0.001).

**Fig. 5:**
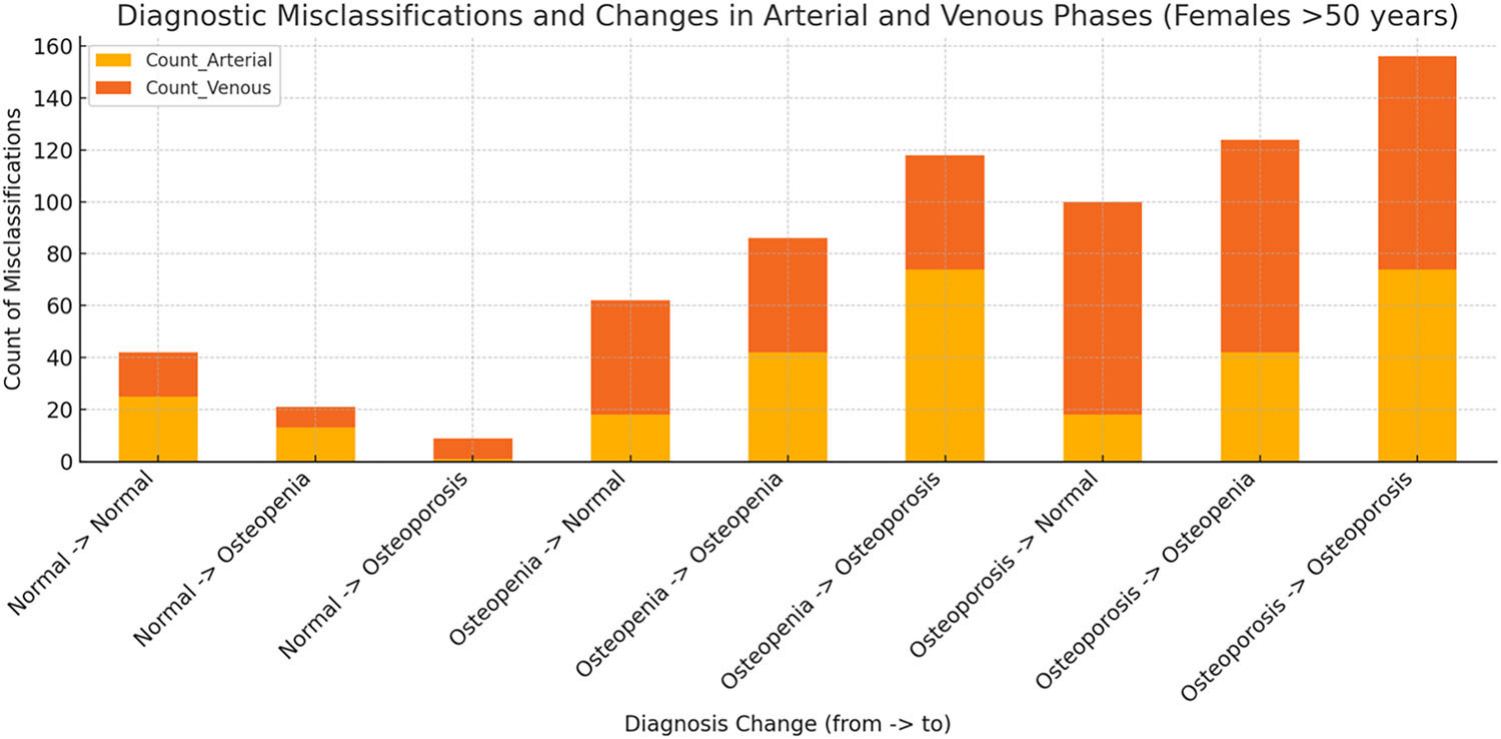
Diagnostic misclassifications in the arterial and venous phase compared to the non-contrast phase in women over 50 years

In the age group of women over 50 years, the most diagnoses changes were observed, with a total diagnosis change rate of 59%. When comparing non-contrast to arterial phases, 16 patients (11%) were incorrectly classified as healthy instead of osteopenic, and two patients (1%) were classified as healthy instead of osteoporotic. Furthermore, 29 patients (21%) were classified as osteopenic instead of osteoporosis. When comparing non-contrast to venous phases, 8 patients (6%) were classified as healthy instead of osteopenic, and 25 patients (18%) were classified as osteopenic instead of osteoporosis (Fig. 5).

**Fig. 6:**
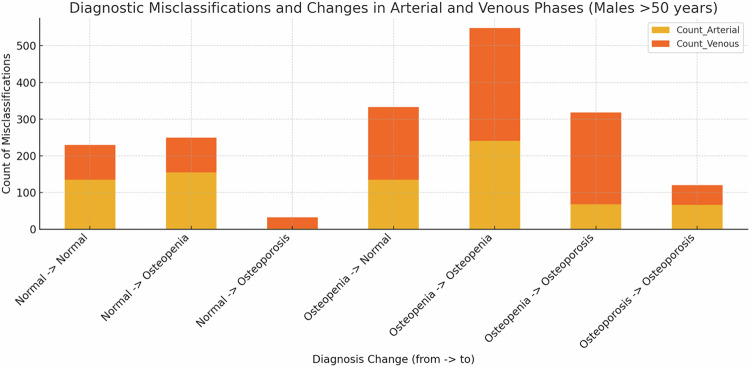
Diagnostic misclassifications in the arterial and venous phase compared to the non-contrast phase in women over 50 years

Similarly, in the age group of men over 50 years, a significant number of diagnostic misclassifications were observed, with 43 patients (11.35%) being misclassified from osteoporosis to osteopenia when comparing non-contrast to arterial phases, and 55 patients (14.52%) when comparing non-contrast to venous phases (*p* < 0.001) (Fig. 6).

## Discussion

Body composition analysis enables opportunistic identification of osteoporosis and osteopenia during routine abdominal CT scans, facilitating prospective clinical evaluations and retrospective population-based assessments to monitor changes over time [20].

In this retrospective study, we examined how varying contrast phases influence the precision of bone density measurements, aiming to optimize imaging protocols for enhanced clinical diagnostics. We have two main findings to report: First, the most significant changes in *T*-scores were observed in patients under 50 years, with increases of +0.71 (99%) at the L1 level and +0.78 (86.7%) at the L1–4 level when comparing unenhanced and early arterial phase scans (*p* < 0.001). Second, of the 597 patients included in the study, 309 (51.76%) experienced a change in diagnosis. Notably, the highest rate of diagnostic changes was observed in women over 50 years, with a total diagnosis change rate of 59%. Specifically, 21% of these women were misclassified as osteopenic instead of osteoporotic when comparing non-contrast to arterial scans. The venous phase showed smaller diagnostic shifts, with 18% of patients being incorrectly classified as osteopenic instead of osteoporotic when comparing non-contrast to venous scans.

Previous studies have examined the impact of tube potential and contrast media (CM) on CT body composition analysis, showing its influence on parameters such as muscle attenuation, skeletal muscle index, and steatotic muscle area in contrast-enhanced CT scans [21].

Further studies have shown that CM significantly affects bone density measurements in CT scans, with notable differences in BMD values between unenhanced and contrast-enhanced scans. These findings emphasize that CM can significantly alter BMD assessments, potentially leading to misleading values, particularly in diagnostic evaluations for osteoporosis [22–25].

While these studies support our results, other studies have demonstrated that automated quantitative measurements of bone, muscle, and fat in contrast-enhanced abdominal CT scans can be accurately correlated with those obtained from non-contrast scans [13, 26–29].

Despite the clear evidence from our study and others that CM significantly affects bone density measurements in individual patients, large cohort studies have often reported less pronounced differences [29]. This apparent discrepancy can be attributed to several factors, primarily the statistical averaging effects inherent in large datasets. When individual patient variations are aggregated in large populations, subtle changes introduced by CM may be diluted, resulting in less dramatic shifts in BMD readings [29]. For instance, previous research has demonstrated that contrast-enhanced CT scans tend to yield higher attenuation values at the L1 vertebral body, on average by approximately eleven HU, compared to unenhanced scans. Despite this measurable increase, the diagnostic accuracy for predicting osteoporosis remains comparable between the two modalities, suggesting that both contrast-enhanced and non-contrast scans may be suitable for initial opportunistic screening purposes [29].

Individual variability in bone density plays a crucial role in diagnostic accuracy, particularly in older populations, where misclassification can result in the absence of treatment or inadequate therapy. The significant influence of CM on individual measurements raises critical clinical concerns. Misclassification of osteoporosis as osteopenia, as observed in our study, can deprive patients of necessary therapeutic interventions, thereby increasing their risk of fractures. Conversely, underestimating BMD in some cases may lead to overtreatment, exposing patients to unnecessary medication risks. Therefore, our study reveals that relying solely on contrast-enhanced scans for bone density evaluation without accounting for phase-specific variation may result in clinically relevant diagnostic misclassifications, especially in subgroups such as postmenopausal women or younger individuals. By systematically comparing unenhanced, early arterial, and venous phases within the same patient cohort, our findings offer a more differentiated perspective on how CM phases impact *T*-score calculations and diagnostic thresholds.

The observation that younger patients under the age of 50 showed more pronounced diagnostic shifts in BMD classification across contrast phases may be explained by their higher proportion of trabecular bone and greater bone marrow content, which likely leads to increased perfusion of CM. As a result, the elevated contrast uptake within the vertebral marrow may artificially raise attenuation values, thereby exaggerating *T*-scores and masking underlying low bone density.

Our study acknowledges several limitations that warrant attention. First, it was intentionally conducted at a single center to minimize variability associated with scanners from different manufacturers or generations. Additionally, this study did not examine the influence of slice thickness despite previous research demonstrating its significant impact on body composition parameters [30].

Furthermore, we used *T*-scores derived from routine abdominal CT scans rather than cut-offs of quantitative computed tomography (QTC) to assess bone density [31]. This choice was influenced by several factors, including the availability and practicality of using *T*-scores in clinical settings and the widespread use of *T*-scores as a standard metric for evaluating bone health in osteoporosis assessments [32]. While QTC directly measures BMD, its implementation in routine clinical practice may be limited due to equipment availability, cost, and additional processing requirements [33]. However, it is essential to acknowledge that *T*-scores may have limitations, particularly in opportunistic CT assessments for osteoporosis. *T*-scores derived from DEXA scans may not capture the same level of detail as QTC regarding regional bone density measurements.

Another limitation of our study is the imbalance in gender and age distribution within the cohort, with a predominance of male patients and a relatively small number of individuals under 50 years of age. This reflects the underlying clinical population, as the included imaging data were primarily obtained from patients undergoing triphasic full-body CT for suspected or confirmed vascular pathologies. While this cohort composition allowed for a reliable and patient-specific assessment of CM effects in a realistic clinical setting, it may limit the generalizability of subgroup analyses, particularly in younger patients.

Moreover, investigating the impact of patient demographics and underlying pathologies on bone density measurements could yield further valuable insights.

A methodological limitation of this study is that only datasets in which both radiologists independently confirmed the anatomical correctness of the segmentation were included in the analysis. Cases with inter-rater disagreement were excluded to ensure a standardized and high-quality ground truth. A consensus-based validation or the involvement of a third reviewer would have represented a more robust methodological strategy. In future studies, we intend to implement a structured protocol aimed at resolving rating discrepancies and systematically investigating their underlying causes.

In conclusion, our study demonstrates that the use of contrast agents in CT scans significantly influences BMD measurements, which can lead to misdiagnosis and misclassification, especially in individual patient cases. Future research should focus on refining CT imaging protocols and developing standardized guidelines to account for CM effects in clinical practice.

## Supplementary information

ELECTRONIC SUPPLEMENTARY MATERIAL

## Abbreviations

BMD: Bone mineral density
CM: Contrast media
CT: Computed tomography
DEXA: Dual-energy X-ray absorptiometry
HU: Hounsfield Units
IQR: Interquartile range
MRI: Magnetic resonance imaging
QTC: Quantitative computed tomography
SD: Standard deviation
