# Supplementary material for: Opportunistic osteoporosis assessment from routine CT—effect of intravenous contrast agents on absolute values, T-scores, and derived classifications in single- and dual-energy CT
Source: Eur Radiol. 2025 Sep 11;36(3):2135–46. doi: 10.1007/s00330-025-11988-1 (PMC12963074; doi:10.1007/s00330-025-11988-1)
Supplement: Supplementary file 1 — ELECTRONIC SUPPLEMENTARY MATERIAL [file 330_2025_11988_MOESM1_ESM.pdf]

# Opportunistic Osteoporosis Assessment from Routine CT - Effect of Intravenous Contrast Agents on Absolute Values, T-Scores, and Derived Classifications in Single- and Dual-Energy CT

## ELECTRONIC SUPPLEMENTARY MATERIAL

**Supplementary Table 1.** Median HU of L1 through the different CT phases

| | TrabecularHU_L1_cont_nat Median | TrabecularHU_L1_cont_nat IQR | TrabecularHU_L1_cont_nat p-value | TrabecularHU_L1_cont_art Median | TrabecularHU_L1_cont_art IQR | TrabecularHU_L1_cont_art p-value | TrabecularHU_L1_cont_ven Median | TrabecularHU_L1_cont_ven IQR | TrabecularHU_L1_cont_ven p-value |
|---|---|---|---|---|---|---|---|---|---|
| **TOTAL** | 127.7 | 95.70-162.60 | <0.01 | 151.2 | 113.37-192.36 | <0.001 | 143.4 | 113.23-175.90 | 0.001 |
| **FEMALES** | 109.9 | 81.72-157.17 | 0.02 | 143.2 | 102.26-191.15 | 0.26 | 133.7 | 101.90-172.12 | 0.09 |
| **MALES** | 131.9 | 100.44-164.55 | 0.24 | 154.6 | 116.12-192.46 | 0.56 | 147.1 | 115.83-176.08 | 0.4 |
| **TOTAL <50 YEARS** | 187.9 | 159.97-215.89 | <0.001 | 220.4 | 184.45-248.00 | <0.001 | 199.1 | 174.12-230.16 | <0.001 |
| **TOTAL >50 YEARS** | 120.0 | 91.57-150.26 | 0.009 | 141.5 | 109.08-181.71 | 0.009 | 135.5 | 107.90-165.48 | 0.009 |
| **FEMALES >50 YEARS** | 104.8 | 75.46-138.85 | <0.001 | 135.2 | 96.14-179.51 | 0.005 | 125.4 | 98.36-158.78 | 0.0003 |
| **MALES >50 YEARS** | 124.3 | 96.08-152.51 | 0.3 | 143.4 | 111.55-183.38 | 0.08 | 137.3 | 112.11-167.43 | 0.2 |

**Supplementary Table 2.** Median HU of L1-4 through the different CT phases

| | TrabecularHU_L1-L4_cont_nat Median | TrabecularHU_L1-L4_cont_nat IQR | TrabecularHU_L1-L4_cont_nat p-value | TrabecularHU_L1-L4_cont_art Median | TrabecularHU_L1-L4_cont_art IQR | TrabecularHU_L1-L4_cont_art p-value | TrabecularHU_L1-L4_cont_ven Median | TrabecularHU_L1-L4_cont_ven IQR | TrabecularHU_L1-L4_cont_ven p-value |
|---|---|---|---|---|---|---|---|---|---|
| **TOTAL** | 121.8 | 89.99-156.93 | <0.001 | 144.8 | 108.25-183.99 | <0..01 | 137.2 | 106.60-169.07 | <0.001 |
| **FEMALES** | 104.7 | 76.73-150.09 | 0.005 | 136.6 | 99.36-180.91 | 0.3 | 124.6 | 97.22-167.20 | 0.0286 |
| **MALES** | 126.1 | 96.13-158.39 | 0.2 | 146.1 | 110.07-184.82 | 0.6 | 141.2 | 110.09-171.95 | 0.271 |
| **TOTAL <50 YEARS** | 180.8 | 147.38-206.04 | <0.001 | 217.1 | 174.69-240.66 | <0.001 | 193.9 | 159.83-215.07 | <0.001 |
| **TOTAL >50 YEARS** | 114.7 | 87.17-144.31 | 0.01 | 136.4 | 102.75-173.68 | 0.02 | 130.6 | 103.70-160.51 | 0.018 |
| **FEMALES >50 YEARS** | 98.7 | 74.20-128.30 | <0.001 | 128.9 | 89.37-167.12 | 0.01 | 118.9 | 95.90-146.56 | <0.001 |
| **MALES >50 YEARS** | 119.6 | 92.12-147.17 | 0.5 | 137.8 | 104.94-174.26 | 0.11 | 133.7 | 106.10-162.07 | 0.350 |

**Supplementary Table 3.** Median Tscore of L1 through the different CT phases

| | TrabecularHU_L1_cont_nat_Tscore Median | TrabecularHU_L1_cont_nat_Tscore IQR | TrabecularHU_L1_cont_nat_Tscore p-value | TrabecularHU_L1_cont_art_TScore Median | TrabecularHU_L1_cont_art_TScore IQR | TrabecularHU_L1_cont_art_TScore p-value | TrabecularHU_L1_cont_ven_TScore Median | TrabecularHU_L1_cont_ven_TScore IQR | TrabecularHU_L1_cont_ven_TScore p-value |
|---|---|---|---|---|---|---|---|---|---|
| **TOTAL** | -2.0 | -2.82--1.29 | <0.001 | -1.5 | -2.44--0.65 | <0.001 | -1.7 | -2.43--0.95 | <0.001 |
| **FEMALES** | -3.2 | -3.98--2.00 | <0.001 | -2.4 | -3.44--1.10 | <0.001 | -2.6 | -3.45--1.60 | <0.001 |
| **MALES** | -1.8 | -2.47--1.14 | 0.0002 | -1.3 | -2.15--0.55 | 0.01 | -1.5 | -2.16--0.90 | 0.001 |
| **TOTAL <50 YEARS** | -0.7 | -1.34--0.08 | <0.001 | -0.005 | -0.73-0.60 | <0.001 | -0.4 | -0.97-0.15 | <0.001 |
| **TOTAL >50 YEARS** | -2.2 | -2.97--1.50 | 0.0109 | -1.8 | -2.56--0.85 | 0.01 | -1.9 | -2.53--1.19 | 0.01 |
| **FEMALES >50 YEARS** | -3.3 | -4.15--2.48 | <0.001 | -2.6 | -3.60--1.41 | <0.001 | -2.8 | -3.54--1.95 | <0.001 |
| **MALES >50 YEARS** | -2.0 | -2.56--1.39 | 0.104 | -1.6 | -2.24--0.74 | 0.68 | -1.7 | -2.23--1.07 | 0.202 |

**Supplementary Table 4.** Median Tscore of L1-4 through the different CT phases

| | TrabecularHU_L1-L4_cont_nat_TScore Median | TrabecularHU_L1-L4_cont_nat_TScore IQR | TrabecularHU_L1-L4_cont_nat_TScore p-value | TrabecularHU_L1-L4_cont_art_TScore Median | TrabecularHU_L1-L4_cont_art_TScore IQR | TrabecularHU_L1-L4_cont_art_TScore p-value | TrabecularHU_L1-L4_cont_ven_TScore Median | TrabecularHU_L1-L4_cont_ven_TScore IQR | TrabecularHU_L1-L4_cont_ven_TScore p-value |
|---|---|---|---|---|---|---|---|---|---|
| **TOTAL** | -2.1 | -2.87--1.48 | <0.001 | -1.7 | -2.60--0.83 | <0.001 | -1.8 | -2.55--1.13 | <0.001 |
| **FEMALES** | -3.4 | -4.11--2.18 | <0.001 | -2.5 | -3.52--1.37 | <0.001 | -2.9 | -3.57--1.73 | <0.001 |
| **MALES** | -1.9 | -2.57--1.27 | 0.0001 | -1.5 | -2.27--0.71 | 0.01 | -1.6 | -2.27--1.00 | 0.0004 |
| **TOTAL <50 YEARS** | -0.9 | -1.56--0.27 | <0.001 | -0.1 | -0.96-0.45 | <0.001 | -0.6 | -1.25--0.05 | <0.001 |
| **TOTAL >50 YEARS** | -2.3 | -3.03--1.65 | 0.017 | -1.9 | -2.73--1.00 | 0.02 | -2.0 | -2.67--1.33 | 0.021 |
| **FEMALES >50 YEARS** | -3.5 | -4.18--2.76 | <0.001 | -2.7 | -3.78--1.73 | <0.001 | -3.0 | -3.61--2.27 | <0.001 |
| **MALES >50 YEARS** | -2.1 | -2.64--1.50 | 0.058 | -1.7 | -2.38--0.93 | 0.57 | -1.8 | -2.35--1.19 | 0.1 |

**Supplementary Table 5.** Median Zscore of L1 through the different CT phases

| | TrabecularHU_L1_cont_nat_ZScore Median | TrabecularHU_L1_cont_nat_ZScore IQR | TrabecularHU_L1_cont_nat_ZScore p-value | TrabecularHU_L1_cont_art_ZScore Median | TrabecularHU_L1_cont_art_ZScore IQR | TrabecularHU_L1_cont_art_ZScore p-value | TrabecularHU_L1_cont_ven_ZScore Median | TrabecularHU_L1_cont_ven_ZScore IQR | TrabecularHU_L1_cont_ven_ZScore p-value |
|---|---|---|---|---|---|---|---|---|---|
| **TOTAL** | -0.5 | -1.13-0.17 | 0.01 | 0.09 | -0.66-0.86 | 0.01 | -0.2 | -0.71-0.52 | 0.01 |
| **FEMALES** | -0.7 | -1.25--0.05 | 0.11 | -0.002 | -0.67-0.70 | 0.66 | -0.3 | -0.68-0.32 | 0.4 |
| **MALES** | -0.4 | -1.09-0.21 | 0.4 | 0.1 | -0.63-0.93 | 0.82 | -0.1 | -0.72-0.56 | 0.7 |
| **TOTAL <50 YEARS** | -0.2 | -0.88-0.59 | 0.02 | 0.6 | -0.16-1.37 | 0.003 | 0.1 | -0.47-0.83 | 0.07 |
| **TOTAL >50 YEARS** | -0.6 | -1.15-0.11 | 0.5 | 0.003 | -0.69-0.80 | 0.35 | -0.2 | -0.73-0.47 | 0.6 |
| **FEMALES >50 YEARS** | -0.7 | -1.28--0.09 | 0.04 | -0.05 | -0.68-0.58 | 0.32 | -0.3 | -0.72-0.25 | 0.2 |
| **MALES >50 YEARS** | -0.5 | -1.10-0.18 | 0.8 | 0.03 | -0.70-0.86 | 0.52 | -0.2 | -0.73-0.55 | 0.9 |

**Supplementary Table 6.** Median Zscore of L1-4 through the different CT phases

| | TrabecularHU_L1-L4_cont_nat_ZScore Median | TrabecularHU_L1-L4_cont_nat_ZScore IQR | TrabecularHU_L1-L4_cont_nat_ZScore p-value | TrabecularHU_L1-L4_cont_art_ZScore Median | TrabecularHU_L1-L4_cont_art_ZScore IQR | TrabecularHU_L1-L4_cont_art_ZScore p-value | TrabecularHU_L1-L4_cont_ven_ZScore Median | TrabecularHU_L1-L4_cont_ven_ZScore IQR | TrabecularHU_L1-L4_cont_ven_ZScore p-value |
|---|---|---|---|---|---|---|---|---|---|
| **TOTAL** | -0.6 | -1.21-0.01 | 0.01 | -0.07 | -0.82-0.73 | 0.01 | -0.3 | -0.80-0.36 | 0.01 |
| **FEMALES** | -0.9 | -1.26--0.24 | 0.05 | -0.2 | -0.72-0.62 | 0.7 | -0.4 | -0.80-0.14 | 0.2 |
| **MALES** | -0.5 | -1.16-0.10 | 0.31 | -0.1 | -0.83-0.78 | 0.8 | -0.2 | -0.80-0.43 | 0.5 |
| **TOTAL <50 YEARS** | -0.4 | -1.05-0.40 | 0.1 | 0.5 | -0.48-1.18 | 0.01 | -0.2 | -0.64-0.58 | 0.2 |
| **TOTAL >50 YEARS** | -0.7 | -1.22-0.00 | 0.7 | -0.1 | -0.84-0.67 | 0.5 | -0.3 | -0.81-0.36 | 0.8 |
| **FEMALES >50 YEARS** | -0.9 | -1.29--0.26 | 0.01 | -0.2 | -0.84-0.59 | 0.4 | -0.5 | -0.81-0.12 | 0.1 |
| **MALES >50 YEARS** | -0.6 | -1.15-0.09 | 0.4 | -0.09 | -0.84-0.72 | 0.6 | -0.2 | -0.80-0.45 | 0.6 |

**Supplementary Table 7.** Median changes in HU of L1 through the different CT phases

| GROUP | art_vs_nat_absolute change | art_vs_nat_Relative_Change | art_vs_nat_CI | art_vs_nat_P_value | ven_vs_nat_absolute change | ven_vs_nat_Relative_Change | ven_vs_nat_CI | ven_vs_nat_P_value |
|---|---|---|---|---|---|---|---|---|
| **TOTAL** | 23.6 | 18.45% | (17.9, 46.7) | <0.001 | 15.7 | 12.30% | (21.30, 13.48) | <0.001 |
| **FEMALES** | 33.3 | 30.31% | (16.8, 29.5) | <0.001 | 23.9 | 21.71% | (25.47, 14.67) | <0.001 |
| **MALES** | 22.7 | 17.22% | (8.96, 53.99) | <0.001 | 15.3 | 11.58% | (15.40, 15.37) | <0.001 |
| **TOTAL <50 YEARS** | 32.5 | 17.26% | (12.9, 42.3) | <0.001 | 11.1 | 5.93% | (10.35, 19.05) | <0.001 |
| **TOTAL >50 YEARS** | 21.7 | 18.05% | (17.6, 24.0) | <0.001 | 15.4 | 12.76% | (21.49, 12.14) | <0.001 |
| **FEMALES >50 YEARS** | 30.4 | 28.99% | (16.2, 34.0) | <0.001 | 20.6 | 19.67% | (25.86, 9.19) | <0.001 |
| **MALES >50 YEARS** | 18.8 | 15.01% | (9.7, 28.1) | <0.001 | 13.2 | 10.54% | (14.14, 10.25) | <0.001 |
| | | | | | | | | |

**Supplementary Table 8.** Median changes in HU of L1-4 through the different CT phases

| GROUP | art_vs_nat_Median | art_vs_nat_Relative_Change | art_vs_nat_CI | art_vs_nat_P_value | ven_vs_nat_Median | ven_vs_nat_Relative_Change | ven_vs_nat_CI | ven_vs_nat_P_value |
|---|---|---|---|---|---|---|---|---|
| **TOTAL** | 23.0 | 18.85% | (12.6, 50.8) | <0.001 | 15.4 | 12.66% | (18.1, 15.7) | <0.001 |
| **FEMALES** | 31.9 | 30.48% | (9.7, 65.0) | <0.001 | 19.9 | 19.02% | (21.2, 18.0) | <0.001 |
| **MALES** | 20.0 | 15.87% | (5.2, 33.4) | <0.001 | 15.1 | 11.94% | (12.8, 8.5) | <0.001 |
| **TOTAL <50 YEARS** | 36.4 | 20.11% | (8.9, 54.5) | <0.001 | 13.1 | 7.22% | (12.9, 16.2) | <0.001 |
| **TOTAL >50 YEARS** | 20.9 | 18.09% | (11.9, 32.3) | <0.001 | 15.1 | 13.08% | (19.3, 13.0) | <0.001 |
| **FEMALES >50 YEARS** | 30.3 | 30.67% | (7.1, 52.9) | <0.001 | 20.2 | 20.50% | (19.6, 16.6) | <0.001 |
| **MALES >50 YEARS** | 18.5 | 15.40% | (6.0, 27.6) | <0.001 | 14.1 | 11.75% | (11.3, 16.7) | <0.001 |

**Supplementary Table 9.** Median changes in Tscores  of L1 through the different CT phases

| GROUP | art_vs_nat_Median | art_vs_nat_Relative_Change | art_vs_nat_CI | art_vs_nat_P_value | ven_vs_nat_Median | ven_vs_nat_Relative_Change | ven_vs_nat_CI | ven_vs_nat_P_value |
|---|---|---|---|---|---|---|---|---|
| **TOTAL** | 0.5 | 22.93% | (0.44, 1.05) | <0.001 | 0.3 | 14.44% | (0.58, 0.28) | <0.001 |
| **FEMALES** | 0.9 | 27.06% | (0.44, 0.77) | <0.001 | 0.6 | 19.38% | (0.67, 0.39) | 0.001 |
| **MALES** | 0.5 | 25.84% | (0.19, 1.12) | <0.001 | 0.3 | 17.57% | (0.32, 0.30) | <0.001 |
| **TOTAL <50 YEARS** | 0.7 | 99.31% | (0.22, 0.95) | <0.001 | 0.3 | 37.46% | (0.26, 0.46) | 0.09 |
| **TOTAL >50 YEARS** | 0.5 | 20.47% | (0.47, 0.46) | <0.001 | 0.3 | 14.18% | (0.54, 0.16) | <0.001 |
| **FEMALES >50 YEARS** | 0.8 | 23.71% | (0.43, 0.89) | <0.001 | 0.5 | 16.09% | (0.68, 0.24) | <0.001 |
| **MALES >50 YEARS** | 0.4 | 20.12% | (0.20, 0.59) | <0.001 | 0.3 | 14.12% | (0.29, 0.21) | <0.001 |

**Supplementary Table 10.** Median changes in Tscores of L1-4 through the different CT phases

| GROUP | art_vs_nat_Median | art_vs_nat_Relative_Change | art_vs_nat_CI | art_vs_nat_P_value | ven_vs_nat_Median | ven_vs_nat_Relative_Change | ven_vs_nat_CI | ven_vs_nat_P_value |
|---|---|---|---|---|---|---|---|---|
| **TOTAL** | 0.4 | 20.09% | (0.42, 1.19) | <0.001 | 0.3 | 14.37% | (0.52, 0.33) | <0.001 |
| **FEMALES** | 0.8 | 24.87% | (0.26, 1.71) | <0.001 | 0.5 | 15.52% | (0.56, 0.47) | 0.001 |
| **MALES** | 0.4 | 21.52% | (0.11, 0.71) | <0.001 | 0.3 | 15.59% | (0.26, 0.19) | <0.001 |
| **TOTAL <50 YEARS** | 0.8 | 86.72% | (0.12, 1.18) | <0.001 | 0.2 | 27.78% | (0.22, 0.36) | 0.09 |
| **TOTAL >50 YEARS** | 0.4 | 19.26% | (0.43, 0.57) | <0.001 | 0.4 | 16.26% | (0.57, 0.25) | <0.001 |
| **FEMALES >50 YEARS** | 0.8 | 22.54% | (0.19, 1.39) | <0.001 | 0.5 | 15.07% | (0.52, 0.44) | <0.001 |
| **MALES >50 YEARS** | 0.4 | 18.81% | (0.13, 0.57) | <0.001 | 0.3 | 14.34% | (0.24, 0.35) | <0.001 |

**Supplementary Table 11.** Median changes in Zscores of L1 through the different CT phases

| GROUP | art_vs_nat_Median | art_vs_nat_Relative_Change | art_vs_nat_CI | art_vs_nat_P_value | ven_vs_nat_Median | ven_vs_nat_Relative_Change | ven_vs_nat_CI | ven_vs_nat_P_value |
|---|---|---|---|---|---|---|---|---|
| **TOTAL** | 0.6 | 115.90% | (0.28, 0.75) | <0.001 | 0.4 | 71.69% | (0.38, 0.27) | <0.001 |
| **FEMALES** | 0.7 | 99.68% | (0.34, 0.76) | <0.001 | 0.4 | 58.41% | (0.39, 0.04) | <0.001 |
| **MALES** | 0.6 | 126.54% | (0.27, 0.69) | <0.001 | 0.3 | 74.30% | (0.39, 0.43) | <0.001 |
| **TOTAL <50 YEARS** | 0.8 | 355.93% | (0.43, 1.18) | 0.0002 | 0.3 | 157.35% | (0.41, 0.23) | 0.07 |
| **TOTAL >50 YEARS** | 0.6 | 101.01% | (0.28, 0.62) | <0.001 | 0.4 | 65.44% | (0.38, 0.38) | <0.001 |
| **FEMALES >50 YEARS** | 0.7 | 93.03% | (0.41, 0.51) | <0.001 | 0.4 | 56.68% | (0.42, 0.20) | <0.001 |
| **MALES >50 YEARS** | 0.5 | 110.06% | (0.26, 0.58) | <0.001 | 0.3 | 70.21% | (0.37, 0.38) | <0.001 |

**Supplementary Table 12.** Median changes in Zscores of L1-4 through the different CT phases

| GROUP | art_vs_nat_Median | art_vs_nat_Relative_Change | art_vs_nat_CI | art_vs_nat_P_value | ven_vs_nat_Median | ven_vs_nat_Relative_Change | ven_vs_nat_CI | ven_vs_nat_P_value |
|---|---|---|---|---|---|---|---|---|
| **TOTAL** | 0.6 | 88.19% | (0.14, 0.97) | <0.001 | 0.4 | 58.76% | (0.30, 0.36) | <0.001 |
| **FEMALES** | 0.7 | 82.06% | (0.31, 1.53) | <0.001 | 0.4 | 49.61% | (0.33, 0.54) | <0.001 |
| **MALES** | 0.5 | 89.01% | (0.05, 1.06) | <0.001 | 0.3 | 63.40% | (0.31, 0.36) | <0.001 |
| **TOTAL <50 YEARS** | 0.9 | 238.19% | (0.36, 1.19) | <0.001 | 0.2 | 61.73% | (0.31, 0.28) | 0.089 |
| **TOTAL >50 YEARS** | 0.6 | 84.71% | (0.13, 0.88) | <0.001 | 0.4 | 59.27% | (0.30, 0.36) | <0.001 |
| **FEMALES >50 YEARS** | 0.7 | 75.71% | (0.30, 1.48) | <0.001 | 0.5 | 48.49% | (0.32, 0.56) | <0.001 |
| **MALES >50 YEARS** | 0.5 | 84.48% | (0.07, 0.87) | <0.001 | 0.3 | 59.18% | (0.34, 0.36) | <0.001 |
